# Prospective study on a fast-track training in psychiatry for medical students: the psychiatric hat game

**DOI:** 10.1186/s12909-020-02304-0

**Published:** 2020-10-19

**Authors:** Anthony Clément, Raphaël Delage, Marie Chollier, Laure Josse, Stéphane Gaudry, Jean-Ralph Zahar, Thierry Baubet, Bertrand Degos

**Affiliations:** 1 Neurology Unit, AP-HP, Avicenne University Hospital, Sorbonne Paris Nord, Bobigny, France; 2 GHU Paris Psychiatrie et Neurosciences, Psychiatry Unit 75G05, Henri Ey Hospital, Paris, France; 3 Department of Infant, Child and Adolescent Psychiatry, AP-HP, Avicenne University Hospital, Sorbonne Paris Nord, Bobigny, France; 4 Social and Political Science Department, University of Chester, Chester, UK; 5 Healthcare simulation center, UFR SMBH, Sorbonne Paris Nord, Bobigny, France; 6 Service de Réanimation Médico-Chirurgicale, AP-HP, Avicenne University Hospital, Sorbonne Paris Nord, Bobigny, France; 7 IAME, UMR 1137, Sorbonne Paris Nord, Paris, France; 8 Service de Microbiologie Clinique et Unité de Contrôle et de Prévention Du Risque Infectieux, AP-HP, Avicenne University Hospital, Sorbonne Paris Nord, Bobigny, France; 9 Université Paris-Saclay, Paris-Sud University, UVSQ, CESP, INSERM, Villejuif, France; 10 Center for Interdisciplinary Research in Biology, Collège de France, CNRS UMR7241/INSERM U1050, Université PSL, Paris, France

**Keywords:** Medical education, Gamified training, Hat game, Learning, Memorization

## Abstract

**Background:**

While medical students are losing interest in lectures in favor of other educational materials, many studies suggest the benefit of active learning, combined with gamified educational tools. The authors developed a psychiatric adaptation of the « Hat Game ». It was hypothesised that this game would increase both knowledge and motivation in medical students toward psychiatric semiology. The aim of the study was to assess the benefit of a Psychiatric Hat Game session for learning psychiatric symptoms in third-year medical students. Student performance was also evaluated at 3 months.

**Methods:**

This gamified fast-track training consists of two teams and each team has to guess as many psychiatric semiology terms as possible using different techniques (i.e. speech, mime). The study involved a pre- and post-evaluation of knowledge (Multiple Choice Questions) and a satisfaction survey. Baseline, post-immediate, and three-months scores were compared by using Friedman analysis for paired samples. Comparisons of mean scores at two different times were performed by using Wilcoxon test for paired samples.

**Results:**

One hundred and sixty-six students were proposed to take part in the study. Among them 129 completed the whole program (response rate = 77.7%). Mean scores measured at the three points in time were significantly different (*p* < 0.001, *N* = 129). Knowledge mean scores were significantly higher after the game than before (+ 28.6%, *p <* 0.001). Improvement was maintained 3 months after the game (+ 18.9%, *p <* 0.001). Satisfaction survey items highlighted that students enjoyed and would recommend this type of gamified training.

**Conclusions:**

The Psychiatric Hat Game improved knowledge of psychiatric semiology in medical students. Results suggest that it is a promising and efficient tool to playfully teach medical semiology, with transferable features, utility and acceptability from one medical field to another. This study contributes to the growing body of knowledge advocating for serious games and gamified training in medical education.

## Background

Medical education is challenging as it must emphasise and lead to the acquisition of theoretical knowledge but also practical skills. For medical students from various cultural groups, gaining information is as important as learning to use, remember and understand it [1]. There is nowadays an increasing trend to move from a classical teacher-centered to a student-centered modality of teaching [2]. Indeed, it is now recognized that this kind of active learning is more beneficial for memorization and improves the performance of the students [3].

Repetition is also thought to increase learning when it occurs incidentally. Some studies showed that intentionally massed repetition might be detrimental to memory whereas non-explicit repetition might be beneficial [4]. Gamified training appears to be a good way to incidentally generate repetition and to provide medical knowledge, especially if several of the four well-known sensory types of inputs (oral/hearing, reading/writing, visual and kinesthetic) are involved [5, 6].

In some medical specialties, such as Neurology, clinical symptoms and signs can easily be orally described and mimed. This property enables the development of active learning methods through play [7]. The hat game is a simple game that takes place over three rounds, in which players have to make their team members guess as many words as possible. Words are usually written on pieces of paper and drawn randomly from a hat. In a recent study, Garcin et al. proposed a neurological version of the traditional Hat Game as an interesting tool to learn neurological semiology [8]. They showed that the Neurological Hat Game (NHG), which is performed in an intrinsically pleasant context, was a playful method to overview and teach a wide range of neurological signs and symptoms. Moreover, the students who participated significantly increased their knowledge scores compared to students who did not take part in the sessions.

Psychiatry is another medical specialty for which an accurate knowledge of semiology is essential in order to establish a diagnosis and ensure adequate treatment. Psychiatric semiology is complex and consists of both old and modern terminologies. Understanding subtle differences in symptomatology is a useful skill to develop for clinical evaluation, differential diagnosis and treatment. Few studies explored methods for learning psychiatric phenomenology, but gamified training and serious games appear to be promising [9–13]. The Hat Game would be an innovative, playful and interesting way to overview psychiatric main symptoms and signs.

For this purpose, we developed a psychiatric version of the Hat Game and assessed its impact in terms of learning and memorization of psychiatric semiology in third-year medical students. To assess the benefit of a Psychiatric Hat Game session for learning psychiatric symptoms, we compared the results of the students to a series of medical questions before, immediately after and 3 months after the game session. Hence, based on the NHG, we demonstrated that the Hat Game is replicable to another medical specialty and in another University.

## Methods

### Design

This observational prospective study was conducted between November 2018 and March 2019 in the Faculty of Medicine Sorbonne Paris Nord. The study was approved by the internal review board of the Faculty of Medicine Sorbonne Paris Nord,authorising this gamified teaching to be included in the medical curriculum for third-year students. Moreover, this work was in accordance with the declaration of Helsinki and al participants gave their oral informed consent to participate.

### Students

The « Psychiatric Hat Game » (PHG) has been integrated into third-year^1^ medical students’ curriculum for Psychiatry (Faculty of Medicine Sorbonne Paris Nord), concomitantly to their first internship in a Psychiatry department. PHG could therefore intervene as a complementary tool to clinical supervising. All the 166 third-year students were included.

In collaboration with the Officer for Healthcare Simulation (LJ), the person in charge of the second cycle of the Medical Studies (SB) and the vice-dean in charge of Pedagogy (JRZ), an online interactive board was generated to propose to all third-year medical students to participate in this teaching. Questionnaires were filled out on tablets. This has been integrated into a revision period of the psychiatric semiology in addition to their usual lecture courses.

Groups of 12–15 students were scheduled to form two teams of 6–7 students. Each group was supervised by a psychiatrist (RD)*.*

### The psychiatric hat game

We used a deck of 63 cards (see Additional file 1). A psychiatric symptom or sign was written on each card. No definition was mentioned on the card. Explanations were given at any time in case of misunderstanding or misinterpretation. This card game was designed by RD and all words were reviewed and validated by one colleague (TB). The task of the game was to guess as many words as possible in a short period (i.e. less than 90 s). The entire game session lasted between 90 and 120 min.

There were three rounds:

1. In the first round, a member of one of the two teams (the clue-giver) had to make his team-mates guess the maximum number of words using any and as many descriptive terms as s/he wanted in less than 90 s. Once this time was elapsed, a member of the opposing team started trying to make his team guess the remaining cards of the deck for another 90 s. Teams alternated until no cards were left or until the end of the dedicated time. The team that won the most cards won the first round.

2. During the second round, the same deck of cards was used and students proceeded in the same way but the clue-giver could give only one word to make his team-mates guess which word was on the card. The team that won the most cards won the second round.

3. The third and final round was similar in principle, but students had to guess the words through a mime. Twenty-two out of the 63 cards were considered too difficult to guess by mime (for instance delusion of filiation or devaluation ideas) and students were allowed to use a small scenario to contextualise the symptom.

At the end of the three rounds, the team that had guessed the most words won. To complete this playful revision of semiology, explanations, exemplifications and supplementary information were provided at any time. Between the first and the second rounds, and at the end of the game, there was an open discussion about symptoms and signs that remained problematic. This debriefing also controlled for potential stereotypes to be formed or expressed, emphasising the complexity of symptoms and experiences. The game was indeed an opportunity to reveal stereotypes. According to the needs of the groups, these were discussed and corrected. The instructor could suggest a more empathetic way of approaching the patient experience.

### Assessment

As a first step, the benefit of this teaching was assessed through 20 Multiple Choice Questions (MCQs) about psychiatric semiology (questionnaires available in Additional file 2). This questionnaire was designed by RD and TB to assess symptoms and diagnosis knowledge. Before the beginning of the game, students had to answer the MCQs. Then, at the end of the day of the game, they were given the same 20 MCQs again, but in a different order. Only fully correct MCQs were counted as valid (1 point per MCQ), and students were given a limited time of 15 min to complete the MCQs. Possible knowledge scores ranged from 0 to 20.

A satisfaction survey (Additional file 3) was completed after the second MCQs session (right after the game). This satisfaction survey is a homemade questionnaire comprising several questions about the interest (positive or negative) of the PHG. It was already used in a recent study [8]. This survey included 8 questions and responses were given according to a 5 choices scale of Likert (1. Strongly agree, 2. Agree, 3. Neutral, 4. Disagree, 5. Strongly disagree). The whole session including the two MCQs’ sessions, the game itself, the teaching period and the satisfaction questionnaire completion lasted about 150 min.

Finally, in order to assess the long-term benefit of such teaching, MCQs were proposed to the same students 3 months later, at the same time as an annual exam. Online interactive board allowed students to answer MCQs and questionnaires on tablets, to avoid missing data and to limit errors when analyzing data.

### Statistical analysis

The data are expressed as mean ± standard deviation (SD) and percentage. We chose to use non parametric tests as data did not follow a normal distribution because of a ceiling effect (20 is the maximum score for the MCQs). The three dependent values of the three times measured were compared with Friedman analaysis. Wilcoxon test for paired samples was used for comparisons between two values. Analyses were performed on IBM SPSS Statistics 23 software (https://www.ibm.com/fr-fr/analytics/spss-statistics-software).

## Results

### Students

**Fig. 1:**
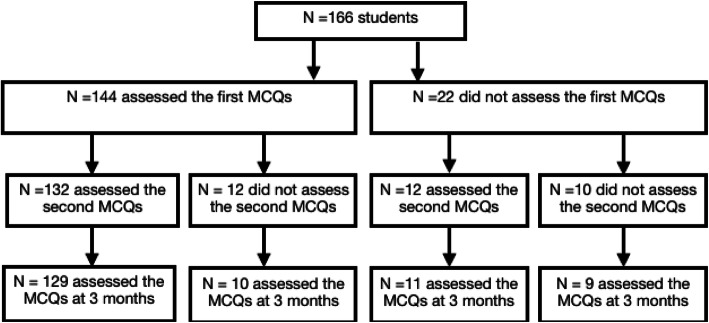
Flowchart. One hundred and sixty-six students participated in the study. One hundred and twenty-nine of them completed all the sessions. MCQs: Multiple Choice Questions

One hundred and sixty-six students were proposed to take part in the study (Fig. 1). Among them, 83 were women (50%). Age ranged from 19 to 35 years old, with a mean at 21.7 ± 2.6. One hundred and fifty-six students played the game. One hundred and thirty-two students underwent both the first and the second test (MCQs). Three of them did not complete the follow-up at 3 months.

### MCQs results

**Fig. 2:**
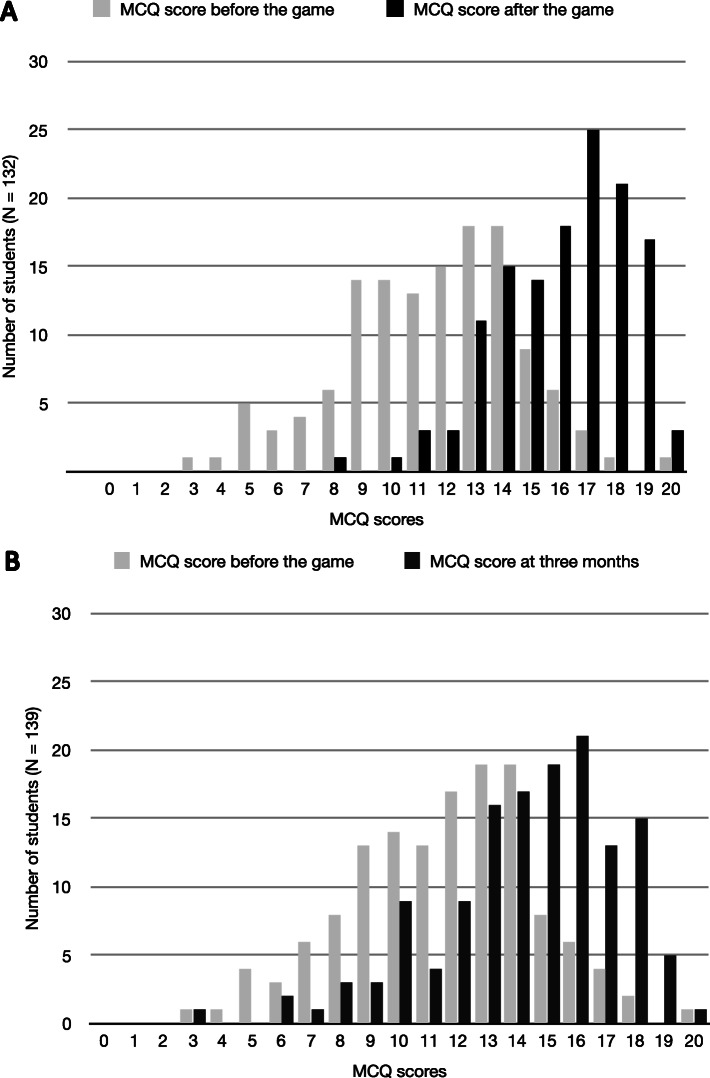
Distribution of students’ knowledge score. **a**. Distribution of students’ knowledge score before (light grey) and just after (black) the Psychiatric Hat Game (PHG). **b**. Distribution of students’ knowledge score before (light grey) and 3 months after (dark grey) the Psychiatric Hat Game (PHG). X axis: scores obtained in MCQs; Y axis: absolute number of students within each subset.

Mean scores measured at the three points in time were significantly different (*p <* 0.001, *N* = 129). Post-test mean MCQs scores significantly increased compared to pre-test (11.5 ± 3.2 [3–20] vs 16.1 ± 2.4 [8–20], *N* = 132, *p <* 0.001, Fig. 2a). At 3 months, among those who participated in the game, the mean score reached 14.3 ± 3.1 [3–20], which was higher than the first scores (11.6 ± 3.2, *p <* 0.001 [3–20], *N* = 139, Fig. 2b). However, these scores were lower than the scores obtained just after the game (14.3 ± 3.1 vs 16.1 ± 2.3, *N* = 140, *p <* 0.001).

### Satisfaction survey

The post-test phase included a satisfaction survey, completed by 144 students. All eight questions obtained a median response of 1. All students found the game playful (129 strongly agreed, 14 agreed, 1 neutral). They also answered that it could help to better understand (121 strongly agreed, 22 agreed, 1 neutral) and remember (108 strongly agreed, 32 agreed, 4 neutral) psychiatric semiology, and that they believed it could be helpful for review in preparation of their annual exam (83 strongly agreed, 50 agreed, 11 neutral). Only two students did not find the modalities appropriate (2 disagreed). Finally, a majority of students answered that the game increased their motivation to learn psychiatric semiology (95 strongly agreed, 29 agreed, 19 neutral and 1 disagreed). These results indicate that the PHG is perceived as an acceptable and useful educational tool for students.

## Discussion

The present study demonstrated the transferability and replicability of the Hat Game to another medical discipline (Neurology but also Psychiatry) and to another institution (Sorbonne University but also Sorbonne Paris Nord) [8]. As expected, the successful application of the Hat Game to psychiatric semiology confirms that the Hat Game is an effective setting for gamified training and that it is easy to create and implement in a teaching or working day. There is no constraint except a sufficient number of student-players.

Here, we applied the Hat Game to teach the psychiatric semiology at the Faculty of Medicine of Sorbonne Paris Nord. The PHG is associated with increased knowledge just after and 3-months after completion. As for the neurological semiology, a satisfaction survey demonstrated that medical students found that the Hat Game is an appreciated tool for teaching semiology such as psychiatric semiology. Compared to the Neurological Hat Game (NHG) [8], almost all of the third-year medical students participated in this teaching game. The high participation rate (77.7% completed the whole program) in third year student cohort does not allow for a comparison group. Furthermore, high scores at pre-test may also relate to the university teaching. A future study could involve several university cohorts with control groups and assess both academic performance in psychiatry examinations and PHG scores. Further studies could also assess whether three or four sessions of a 2 h gamified training could be a cost-efficient fast-track training, and be integrated into university curricula and medical staff routine. Team work is also documented as a way to facilitate competitiveness and increase the student’s motivation and satisfaction [10, 14] .

As shown for the NHG, gamified teaching is a facilitative method for learning that probably involves mesocorticolimbic circuits [15, 16]. Teaching and learning in a pleasant and non stressful environment appears to improve memorization [8, 17–20]. In addition to short-term memory benefits, the MCQs results at 3 months suggest that this playful method could facilitate long-term retention. This long-term benefit is however lower than the post-immediate one. A decrease in scores after several weeks in comparison with the post-immediate is recurrently described in the literature [21, 22], indicating the potential need for booster sessions. Similar enthusiastic teaching was already applied by Roze et al., demonstrating its efficiency in facilitating long term memory [23].

These results are consistent with the literature data suggesting that serious games are useful when associated with standard lecturing [24]. Other playful methods have been proven efficient to increase students’ knowledge in psychosis or dementia [9, 12]. Techniques, such as simulation, could enable students to be more confident in diagnosing and managing psychiatric disorders [25, 26]. Developing such educational materials seems all the more important as learning psychiatry plays an important role in destigmatising this discipline with future health professionals. Moreover, it would encourage “person-centered” approaches to medical practice [27–29].

Despite positive results, our open-labeled study presented methodological limitations. Further research could focus on a randomised controlled trial or a study-design where the control group would attend a classic lecture (or read a textbook) for the same amount of time that the experimental group would participate in a PHG session. An interesting point would also be to apply again at 3 months the satisfaction survey to verify if the students found this educational approach useful. Booster sessions could also help enhance the benefit of the game. It would be interesting to perform a multicenter study to confirm the external validity of our findings but differences in the organization of the medical studies between faculties could be a problem. Finally, further studies are needed to confirm the effects on short-term and long-term memory retention. Another way to validate this training could be to show that this increase in knowledge translates in other assessments, such as Objective Structured Clinical Exams (OSCE).

A further challenge with the PHG relies on stereotypes and the development of mental health stigma in young medical students [30–32]. Service users, researchers and health professionals have highlighted the deleterious impact of stigma and caricatured portrayal might contribute to erroneous and stigmatizing representations [33, 34]. To address these concerns, two strategies have been considered: including mental health stigma questionnaires in the pre/post evaluation design [35, 36] and adapting and piloting efficient interventions retrieved from the stigma-reducing literature. The PHG could be i) co-facilitated with a person living with a mental health difficulty or complemented with a testimony as direct (in-person) and indirect contact (video) have been documented and proven to be efficient in medical students [37, 38]; ii) include perspective-taking exercises and/or debriefs and anti-stigma training in the medical curriculum [39, 40]; iii) include reflexive exercises to encourage medical students to identify self-stigma and seek help when appropriate, as medical students are vulnerable to mental health difficulties and related stigma [41, 42]. Since mental health stigma in medical professions is under-researched in France, both medical students and service users would benefit from further research about integrative training addressing stigma, participants’ concerns and ensuring knowledge acquisition.

In conclusion, the Psychiatric Hat Game seems to be a very interesting and enjoyable educational tool, which improved medical students’ performance/knowledge of psychiatric signs and symptoms in both the short- and long-term. The Psychiatric Hat Game may present an interesting and promising application for learners in other medical disciplines and international settings.

## Supplementary information

**Additional file 1.** List of the 63 symptoms and signs used in the game and their indicative English translation. Twenty-two of them were found too difficult to guess by mime at round 3. For these items, students were told to play them as theater stage. They were written in red on the cards but appear here in italic.

**Additional file 2.** Multiple Choice Questions assessing students’ knowledge of psychiatric symptoms and signs. Medical students were asked to answer this questionnaire before and right after the game session. MCQs were also completed 3 months after the session.

**Additional file 3.** Satisfaction survey completed by the students after the game session.

## Data Availability

The datasets used and analysed during the current study are available from the corresponding author on reasonable request.

## Abbreviations

NHG: Neurological Hat Game
PHG: Psychiatric Hat Game
MCQ: Multiple Choice Question
OSCE: Objective Structured Clinical Exam

## Footnotes

1 In France, the first two years of medical studies are dedicated to the learning of anatomy, physiology and biology. The third year focuses on medical semiology. Students are taught to perform a clinical exam, recognize different symptoms, and organize them into syndromes.
