# Supplementary material for: Prospective study on a fast-track training in psychiatry for medical students: the psychiatric hat game
Source: BMC Med Educ. 2020 Oct 19;20:373. doi: 10.1186/s12909-020-02304-0 (PMC7574431; doi:10.1186/s12909-020-02304-0)
Supplement: Supplementary file 1 — Additional file 1. List of the 63 symptoms and signs used in the game and their indicative English translation. Twenty-two of them were found too difficult to guess by mime at round 3. For these items, students were told to play them as theater stage. They were written in red on the cards but appear here in italic. [file 12909_2020_2304_MOESM1_ESM.docx]

| **French symptoms and signs** | **English translation** |
| --- | --- |
| Aboulie | Aboulia |
| Abstinence | Abstinence |
| Akathisie | Akathisia |
| Anhédonie | Anhedonia |
| Asthénie | Asthenia |
| Attitude d’écoute | Listening attitude (from patient) |
| *Autodévalorisation* | *Self-downing, self-devaluation* |
| Automutilation | Self-mutilation, non suicidal self injury (NSSI) |
| Barrage | Thought blocking |
| Bradykinésie | Bradykinesia |
| Bradyphémie | Bradyphemia |
| Catalepsie | Catalepsy |
| Clinophilie | Clinophilia |
| Compulsion | Compulsion |
| Conversion | Conversion |
| *Délire de filiation* | *Delusion of filiation* |
| *Dépersonnalisation* | *Depersonalization* |
| Désinhibition | Disinhibition |
| *Diffluence* | *Diffluent speech* |
| Discordance idéo-affective | Inappropriate affect, Lack of congruence between ideas/speech and affects/emotions |
| Douleur morale | Mental pain |
| Dysmorphophobie | Dysmorphophobia |
| *Echolalie* | *Echolalia* |
| Enurésie | Enuresis |
| Evitement | Avoidance |
| *Fuite des idées* | *Flight of ideas* |
| *Hallucination intrapsychique* | *Internal hallucination* |
| *Hermétisme* | *Hermetism* |
| Hypermimie | Hypermimia |
| Hypersyntonie | Hypersyntonia |
| *Idées de référence* | *Ideas of reference* |
| *Idées de ruine* | *Ideas of ruin* |
| Incurie | Carelessness, self-neglect (mostly in relation to hygiene) |
| Labilité émotionnelle | Emotional lability |
| *Logorrhée* | *Logorrhea* |
| Ludisme | Ludism, playfulness |
| Maniérisme | Mannerism |
| *Mécanisme interprétatif* | *Interpretative mechanism* |
| Négativisme | Negativism |
| *Néologisme* | *Neologism* |
| Obnubilation | Obnubilation |
| Obséquiosité | Obsequiousness |
| *Paralogisme* | *Paralogism* |
| Pathomimie | Pathomimia |
| *Pensée magique* | *Magical thinking* |
| *Phobie d’impulsion* | *Phobia of committing impulsive act* |
| Potomanie | Potomania |
| Quérulence | Querulousness |
| *Rationalisme morbide* | *Morbid rationalism* |
| Reviviscence traumatique | Traumatic flashbacks, revivification episodes of traumatic experience |
| Ruminations | Rumination |
| *Schizophasie* | *Schizophasia, unintelligible speech,*  *word salad* |
| *Sentiment de culpabilité* | *Feeling of guilt* |
| *Sentiment d’incurabilité* | *Feeling of incurability* |
| Soliloquie | Soliloquy |
| Stéréotypie | Stereotypy |
| Stupeur | Stupor |
| *Syndrome de Cotard* | *Cotard syndrome* |
| Tachypsychie | Racing thoughts |
| Terreur nocturne | Night terror |
| Théâtralisme | Theatricality |
| *Thème de persécution* | *Persecutory theme* |
| *Thème mystique* | *Mystical theme* |
