# Supplementary material for: Prospective study on a fast-track training in psychiatry for medical students: the psychiatric hat game
Source: BMC Med Educ. 2020 Oct 19;20:373. doi: 10.1186/s12909-020-02304-0 (PMC7574431; doi:10.1186/s12909-020-02304-0)
Supplement: Supplementary file 2 — Additional file 2. Multiple Choice Questions assessing students’ knowledge of psychiatric symptoms and signs. Medical students were asked to answer this questionnaire before and right after the game session. MCQs were also completed 3 months after the session. [file 12909_2020_2304_MOESM2_ESM.docx]

**Q°1** Are you a man or a woman?

**Q°2** How old are you?

**Q°3** Negativism:

A. is a tendency to devalue the future

B. is an attitude of active opposition

C. is a painful remembrance

D. is a tendency of systematic criticism

**Q°4** Listening attitude:

A. refers to a listening posture without external sound stimulation

B. refers to the behavior of a patient asking for advice

C. refers to a pathological submission to authority

D. refers to the inversion of the therapeutic relationship from a patient

**Amongst these symptoms: ludism, neologism, ideas of ruin, magical thinking,**

**Q°5** Which one is a symptom of schizophrenic disorganisation:

A. ludism

B. neologism

C. ideas of ruin

D. Magical thinking

**Q°6** Which one is a depressive symptom:

A. Ludism

B. Neologism

C. Ideas of ruin

D. Magical thinking

**Q°7** Which one is specific to Obsessive Compulsive Disorder:

A. Ludism

B. Neologism

C. Ideas of ruin

D. Magical thinking

**Q°8** Conversion Disorder is:

A. A sudden change of thinking or feeling

B. A perceived constraint to achieve mathematical operations

C. A physical symptom originated an assume psychical cause

D. A pathological change of religion

**Q°9** Among these symptoms, which one is not a theme of delusion?

A. Filiation

B. Interpretation

C. Mysticism

D. Persecution

**Q°10** Which symptom is not a part of the depressive syndrome?

A. Bradypsychia

B. Feeling of incurability

C. Asthenia

D. Hypersyntony

**Amongst these symptoms: tachypsychia, aboulia, stereotypy, bradyphemia, dysmorphophobia**

**Q°11** Which one is a manic symptom:

A. Tachypsychia

B. Aboulia

C. Stereotypy

D. Bradyphemia

E. Dysmorphophobia

**Q°12** Which one is a symptom of schizophrenia?

A. Tachypsychia

B. Aboulia

C. Stereotypy

D. Bradyphemia

E. Dysmorphophobia

**Q°13** Which one is a symptom of anorexia nervosa?

A. Tachypsychia

B. Aboulia

C. Stereotypy

D. Bradyphemia

E. Dysmorphophobia

**Q°14** Enuresis is:

A. A layer of fine hairs

B. A loss of urine

C. A speech difficulty

D. A lack of hygiene

**Amongst these symptoms: ideas of reference, avoidance, traumatic flashbacks, stupor,**

**Q°15** Which one is atypical in Post Traumatic Stress Disorder?

A. Ideas of reference

B. Avoidance

C. Traumatic flashbacks

D. Stupor

**Q°16** Which one is typical in Specific Phobia?

A. Ideas of reference

B. Avoidance

C. Traumatic flashbacks

D. Stupor

**Q°17** Hermetism is:

A. A hair abnormality secondary to anorexia nervosa

B. A speech lacking clarity

C. A social flight behavior

D. A tendency to seclusion and confinement

**Q°18** Amongst these symptoms, which one is not typical of the Obsessive Compulsive Disorder?

A. Phobia of committing impulsive acts

B. Arithmomania

C. Checking ritual

D. Dysmorphophobia

**Q°19** Clinophilia refers to:

A. A sexual disorder

B. An obsession with hygiene

C. A tendency to stay in bed

D. A tendency to collectionism

**Q°20** Which one of these symptoms is a part of the disorganisation syndrome in schizophrenia?

A. Logorrhea

B. Persecution

C. Anhedonia

D. Thought blocking

**Q°21** Pathomimia refers to:

A. Theatrical facial expressions

B. The invention of a factitious disorder

C. Poor facial expressions

D. Slowed gestures

**Q°22** Speaking alone is called:

A. Monoloquy

B. Soliloquy

C. Monophonia

D. Soliphonia
