# Supplementary material for: Prospective study on a fast-track training in psychiatry for medical students: the psychiatric hat game
Source: BMC Med Educ. 2020 Oct 19;20:373. doi: 10.1186/s12909-020-02304-0 (PMC7574431; doi:10.1186/s12909-020-02304-0)
Supplement: Supplementary file 3 — Additional file 3. Satisfaction survey completed by the students after the game session. [file 12909_2020_2304_MOESM3_ESM.docx]

The Psychiatric Hat Game (PHG) is fun:

1. Strongly agree

2. Agree

3. Neutral

4. Disagree

5. Strongly disagree

The PHG has been helpful for you to better understand the psychiatric signs and symptoms:

1. Strongly agree

2. Agree

3. Neutral

4. Disagree

5. Strongly disagree

The PHG helped you to better remember psychiatric signs and symptoms

1. Strongly agree

2. Agree

3. Neutral

4. Disagree

5. Strongly disagree

The PHG has been helpful for revising your upcoming exam:

1. Strongly agree

2. Agree

3. Neutral

4. Disagree

5. Strongly disagree

The PHG has increased your motivation to learn psychiatric semiology:

1. Strongly agree

2. Agree

3. Neutral

4. Disagree

5. Strongly disagree

The PHG modalities are appropriate (duration, play followed by discussion time and debugging):

1. Strongly agree

2. Agree

3. Neutral

4. Disagree

5. Strongly disagree

The PHG must be replicated in the future:

1. Strongly agree

2. Agree

3. Neutral

4. Disagree

5. Strongly disagree

The PHG should be extended to other medical specialties:

1. Strongly agree

2. Agree

3. Neutral

4. Disagree

5. Strongly disagree
